# Association of religiosity and spirituality with quality of life in patients with cardiovascular disease: a systematic review

**DOI:** 10.1007/s11136-018-1906-4

**Published:** 2018-06-11

**Authors:** Hawa O. Abu, Christine Ulbricht, Eric Ding, Jeroan J. Allison, Elena Salmoirago-Blotcher, Robert J. Goldberg, Catarina I. Kiefe

**Affiliations:** 1 Department of Quantitative Health Sciences, University of Massachusetts Medical School, 368 Plantation Street, Worcester, MA 01605 USA; 2 Centers for Behavioral and Preventive Medicine, The Miriam Hospital, Providence, RI 02903 USA; 3 Department of Epidemiology, Brown School of Public Health, Providence, RI 02903 USA; 4 Warren Alpert School of Medicine & School of Public Health, Brown University, Providence, RI 02903 USA

**Keywords:** Religiosity, Spirituality, Quality of life, Global QOL, Health-related QOL, Cardiovascular disease

## Abstract

**Purpose:**

This review systematically identified and critically appraised the available literature that has examined the association between religiosity and/or spirituality (R/S) and quality of life (QOL) in patients with cardiovascular disease (CVD).

**Methods:**

We searched several electronic online databases (PubMed, SCOPUS, PsycINFO, and CINAHL) from database inception until October 2017. Included articles were peer-reviewed, published in English, and quantitatively examined the association between R/S and QOL. We assessed the methodological quality of each included study.

**Results:**

The 15 articles included were published between 2002 and 2017. Most studies were conducted in the US and enrolled patients with heart failure. Sixteen dimensions of R/S were assessed with a variety of instruments. QOL domains examined were global, health-related, and disease-specific QOL. Ten studies reported a significant positive association between R/S and QOL, with higher spiritual well-being, intrinsic religiousness, and frequency of church attendance positively related with mental and emotional well-being. Approximately half of the included studies reported negative or null associations.

**Conclusions:**

Our findings suggest that higher levels of R/S may be related to better QOL among patients with CVD, with varying associations depending on the R/S dimension and QOL domain assessed. Future longitudinal studies in large patient samples with different CVDs and designs are needed to better understand how R/S may influence QOL. More uniformity in assessing R/S would enhance the comparability of results across studies. Understanding the influence of R/S on QOL would promote a holistic approach in managing patients with CVD.

**Electronic supplementary material:**

The online version of this article (10.1007/s11136-018-1906-4) contains supplementary material, which is available to authorized users.

## Introduction

Cardiovascular disease (CVD) is the leading cause of morbidity and mortality worldwide, with an estimated 17.7 million deaths from CVD in 2015 [1]. Patients with CVD experience numerous physical symptoms including fatigue, dyspnea, or chest pain, which affects their physical, emotional, and social well-being with significant impairment in quality of life (QOL) [2]. While current strategies for the management of patients with CVD are designed to reduce morbidity and prolong survival, treatment should also be focused on improving patient’s QOL by reducing their symptoms, optimizing life’s daily functions, and overall well-being [2, 3]. Cardiac rehabilitation programs involving lifestyle modification, psychological interventions, education, and counseling have been shown to limit the adverse physiologic and psychologic effects associated with cardiac illness and enhance patient’s QOL [4].

The World Health Organization defines QOL as ‘a broad ranging concept affected in a complex way by the person’s physical health, psychological state, level of independence, social relationships and their relationship to salient features of their environment’ [5]. Global QOL broadly assesses the overall impact of disease on an individual’s life, while health-related QOL (HRQOL) focuses on the impact of health conditions and their symptoms on patients’ well-being [6]. Prior studies in patients with CVD have identified QOL as a sensitive patient-reported outcome measure of various intervention strategies [7], as an independent determinant of survival among patients with CVD [8, 9], and have reported a gradual decline in QOL with increasing number of CVD risk factors present [10, 11].

There is no consensus as to the definitions of “religiosity” or “spirituality.” For purposes of this systematic review, we have utilized working definitions of religiosity/spirituality (R/S) used in the prior literature [12, 13]. Religious practices and spiritual beliefs influence coping mechanisms in dealing with various chronic illnesses [14–16]. In many patients with CVD, R/S are important and highly personal aspects of their disease experience and provide vital strategies for coping [17]. Studies on the relationship between R/S and QOL among patients with various forms of CVD have, however, demonstrated mixed results. While several reviews have examined factors associated with QOL in patients with CVD [18, 19], the association between R/S and global or HRQOL among patients with CVD has received limited attention.

The objective of this systematic review is to summarize and critically appraise available evidence on the association between R/S and QOL in patients with CVD. Understanding this relationship may help in developing intervention strategies to promote spiritual well-being and to optimize QOL in patients with chronic CVD.

## Methods

This review was registered in the international prospective registry of systematic reviews PROSPERO (identification #: CRD42017076970) and conducted in accordance with the Preferred Reporting Items for Systematic Reviews and Meta-Analyses (PRISMA) guidelines [20].

### Search strategy

We searched four electronic databases (PubMed, SCOPUS, PsycINFO, and Cumulative Index to Nursing and Allied Health Literature (CINAHL)) from database inception with no constraints on publication year. All searches were conducted between September 15, 2017 and October 20, 2017. Two reviewers (H.O.A and C.U) worked in conjunction with two medical research librarians to create a search algorithm that used Medical Subject Headings (MeSH) terms and key words related to “religiosity” and “spirituality” (including related terms religious, religiousness, and spiritual) in combination with “quality of life” (and its associated synonyms HRQOL and well-being), and “cardiovascular disease” (with related terms acute myocardial infarction, acute coronary syndrome, congenital heart disease, rheumatic heart disease, heart failure, and cardiac surgery). The reference sections of eligible full-text articles were examined to identify additional studies suitable for inclusion. The full search algorithm is presented in an electronic supplementary material (Online Resource 1).

### Eligibility criteria

We included only full-text peer-reviewed articles published in English that provided quantitative data with no restriction on study design (observational, randomized controlled trials). Qualitative studies, case reports, and reviews were excluded. Studies of patients with various forms of CVD including heart failure, acute myocardial infarction, coronary heart disease, atrial fibrillation, and congenital heart disease were included. The study population included patients of all ages, at different stages of their care (in-hospital, community dwelling, rehabilitation), and those who received any form of cardiac treatment (medical or surgical). Studies were included if they specifically assessed patient’s R/S and assessed either patient’s HRQOL, global QOL, or disease-specific QOL as the primary study outcome. The included studies had to assess the direct relationships between R/S and QOL, and studies that examined R/S and QOL as potential mediators were excluded from further evaluation.

### Review process

Study eligibility was assessed by an initial review of the article title followed by a review of the abstract. Full-text publications were subsequently retrieved of eligible articles and those that met our inclusion criteria were retained for data abstraction. One reviewer (H.O.A) independently conducted the reviews, while another reviewer (E.D) determined the appropriateness of final article inclusion. The two reviewers (H.O.A and E.D) met weekly to discuss the eligibility of included studies, and the inter-rater agreement between both reviewers was calculated using Cohen’s Kappa statistic [21]. Any discrepancies related to article eligibility were discussed and resolved with reference to the explicit eligibility criteria. If no consensus was reached, a co-author (C.U) provided final judgement about article inclusion or exclusion.

### Data extraction

A standardized form was used to obtain relevant information from eligible articles including publication date, authors, country of origin, study design, recruitment, completion rates, sample size, and baseline characteristics of the study population. Detailed information was obtained regarding the measures of QOL and R/S including the scale used, number of items, dimensions captured, and the scoring system. The statistical measure(s) of association between R/S and the respective QOL measures were obtained. Two authors (H.O.A and E.D) completed the data extraction process independently.

### Study quality assessment

The methodological quality of identified studies was critically appraised using a revised version of the Downs and Black quality rating scale [22]. The Downs and Black scale was originally developed to assess quality in clinical trials with a checklist consisting of 27 items and a maximum score of 32 points. Similar to prior systematic reviews [23, 24], we revised the scale to allow for the assessment of observational studies. The modified checklist comprised 13 items with a maximum score of 14 for assessing cross-sectional studies, and 18 items with a maximum score of 19 for longitudinal studies. For each study, a quality score (in percentages) was obtained by dividing the number of points earned by the total number the study was eligible to receive based on appropriate reporting of study objectives, methods, results, and validity. Given the limited number of studies identified in this review, no exclusions were based on the quality assessment. Results of the methodological quality assessment are available in an electronic supplementary material (Online Resource 2).

### Data synthesis

The included studies were too heterogeneous for a meta-analysis to be conducted. Heterogeneity between studies was observed in the varying approaches used to assess R/S, ranging from the different instruments used across studies to multiple dimensions of R/S examined; these issues have been acknowledged in prior systematic reviews [25, 26]. We provide a qualitative synthesis of the results obtained from the studies identified in our review.

## Results

### Study selection

**Fig. 1:**
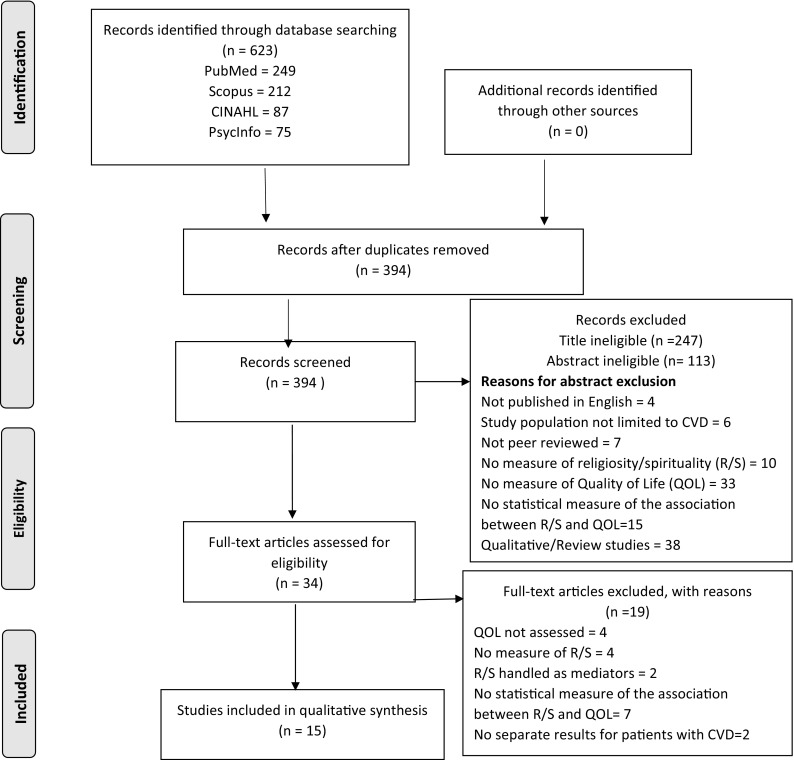
Flow diagram for systematic review methodology in accordance with PRISMA guidelines

Our database search retrieved 623 potentially relevant studies, from which 229 duplicates were removed. Following title and abstract review, 360 articles were excluded leaving 34 full-text articles to be screened for eligibility. We excluded 19 full-text articles that did not measure QOL or R/S, did not statistically assess the association between R/S and QOL, or treated R/S or QOL as mediators. The remaining 15 articles were included in this review. Agreement between the two reviewers on the selection of full-text articles was high (Cohen’s *κ* 0.90). No eligible articles were identified from the reference lists of included studies. Of the 15 publications in this review, four used data derived from a single cohort study [27–30], while two articles used data from another cohort investigation [31, 32]. Publications using data from the same cohort study were considered individually due to their varying study objectives and findings. Detailed results of our screening process are presented in Fig. 1.

### Description of included studies

#### Study design and setting

The fifteen studies included in this review were published between 2002 and 2017; most were conducted in the US (*n* = 12, [27–38]) while others were carried out in Greece (*n* = 1, [39]), Iran (*n* = 1, [40]), and Korea (*n* = 1, [41]). All identified studies were observational; two-thirds used a cross-sectional design (*n* = 9, [30, 33–35, 37–41]), while six studies used a longitudinal design [27–32]. Study follow-up periods ranged from 3 months [27–29, 32] to 2 years [31]. Study sample sizes ranged from 58 to 163 patients with varying manifestations of CVD.

#### Patient characteristics

In all studies except for one, patients were typically middle aged or older with the mean age at the time of study enrollment ranging from 53 to 67 years. The patient populations were predominantly male (range 48–79%) and married (range 50–91%). The only exception was a study that included adult patients with congenital heart disease [41]; the mean age of these patients at study enrollment was 26.5 years and only 10.6% were married. Patient’s racial distribution was reported only in US-based studies with a predominance of non-Hispanic Whites (range 47–100%). Nine publications [27–33, 39, 40] reported religious affiliation. In the Greek study [39], all participants were Orthodox Christians; while in the Iranian study [40], all participants were Muslims. In the seven US-based studies that provided data on religious affiliation [27–33], most participants were Protestants (range 62–72%) or Catholics (range 16–29%). Eleven of the fifteen studies enrolled patients with heart failure [27–30, 33–38, 40]), and the average time since diagnosis varied between 6 months and 6.5 years. One study [39] enrolled patients with varying diagnoses of CVD. Other studies included patients with a diagnosis of myocardial infarction [31, 32] and congenital heart disease [41]. Table 1 provides a detailed description of studies included in this systematic review.

**Table 1:** Summary of full-text articles included in this systematic review

Authors (pub. year) Country [reference]	Study design	Sample size	Participants	Type of cardiovascular disease; time since diagnosis		Religiosity and/or spirituality measure	Quality-of-life measure		Statistical analysis	Major findings
Park et al (2011) USA [27]	Longitudinal study (3 months duration)	111 enrolled; 101 followed up	60.3% men Mean age (SD) = 66.7 years (11.0) 56% Caucasian 39% African American 10% Latino 5% Native American 60% married 67% protestant 16% Catholic 1% Jewish 9% no religious affiliation	Heart failure; mean length of diagnosis = 6.5 years SD = 5.6 years		1. Religious struggle measured by the religious strain scale 2. Religious comfort measured with the daily spiritual experience scale 3. Religious identification (measured at 1 and 3 months respectively)	1. HRQOL SF-12 2. MLWHFQ (measured at baseline and 3 months respectively)		1. Correlation Analysis 2. Hierarchical regression analysis	1. At baseline, religious struggle measured was not significantly correlated with physical impairment (*r* = 0.13, *p* > 0.05), as well as the physical (*r* = − 0.11, *p* > 0.05) nor mental (*r* = 0.06, *p* > 0.05) components of HRQOL 2. Religious struggle measured at baseline was not significantly correlated with physical impairment, (*r* = 0.20 *p* < 0.10) as well as the physical (*r* = − 0.14, *p* > 0.05) nor mental (*r* = − 0.15, *p* > 0.05) components of HRQOL measured at 3 months 3. Religious struggle at baseline did not predict change in QOL comparing 3 months to baseline
Park et al (2014) USA [28]	Longitudinal study (3 months duration)	111 enrolled; 101 followed up	60.3% men Mean age (SD) = 66.7 years (11.0) 56% Caucasian 39% African American 10% Latino 5% Native American 67% Protestant 16% Catholic 1% Jewish 9% no religious affiliation Marital status not reported	Heart failure; mean length of diagnosis = 6.5 years SD = 5.6 years		1. Religious strain scale 2. BMMR/S (measured at 1 and 3 months, respectively)	1. MLWHFQ 2. HRQOL-SF12 (measured at 1 and 3 months, respectively)		1. Correlation analysis 2. Hierarchical longitudinal regression	1. Only one dimension of R/S (i.e., daily spiritual experience) at 1 month was significantly correlated with physical well-being at 3 months. (*r* = − 0.29, *p* < 0.05) 2. Belief in afterlife at 1 month was negatively correlated with mental HRQOL at 3 months (*r* = − 0.21, *p* < 0.05) 3. In longitudinal hierarchical models, no dimensions of R/S predicted physical well-being
Sacco et al (2014) USA [29]	Longitudinal study (3 months duration)	111 enrolled 103 followed up	60.3% men Mean age (SD) = 66.7 years (11.0) 56% Caucasian 39% African American 10% Latino 5% Native American 67% Protestant 16% Catholic 1% Jewish 9% no religious affiliation Marital status not reported	Heart failure; mean length of diagnosis = 6.5 years SD = 5.6 years		Open-ended questions on coping with illness	HRQOL-SF12		Correlation Analysis	1. Religion/Spirituality was not significantly correlated with the mental (*r* = 0.14, *p* > 0.05) nor physical (*r* = -0.11, *p* > 0.05) components of HRQOL measured at baseline 2. Religion/Spirituality measured at baseline was significantly correlated with only the physical component of HRQOL measured 3 months after enrollment (*r* = 0.20, *p* < 0.05)
Park & Sacco (2017) USA [30]	Cross-sectional study	111	60.3% men Mean age (SD) = 67 years (11.4) 56% Caucasian 39% African American 10% Latino 5% Native American 61% married 67% protestant 17% Catholic 9% no religious affiliation < 1% Jewish	Heart failure; mean length of diagnosis = 6.5 years SD = 5.6 years		1. Spiritual desires, constraints, and needs questionnaire 2. The Daily Spiritual Experience subscale of the BMMR/S	HRQOL-SF12		Subgroup regression analysis according to patients who desired spiritual attendance or not	1. In patients who desired spiritual attendance, spiritual constraint was associated with poorer physical quality of life (*β* = −0.39, *p* < 0.01) 2. In patients who did not desire spiritual attendance, having their spiritual needs met was associated with higher mental (*β* = 0.24 *p* < 0.10) and physical quality of life (*β* = 0.29, *p* < 0.05)
Trevino et al (2014) USA [31]	Longitudinal study (2 years duration)	Full sample = 105 Analytic sample = 43	79% men Mean age (SD) = 60.2 years (10.9) 100% White 91% married 72% protestant	First time Myocardial Infarction patients or Post-Coronary Artery bypass Surgical		1. The Religious Coping Activities Scale 2. The Religiosity Measure (measured at baseline, 1 year, and 2 years)	QLMI (measured at baseline, 1 year, and 2 years)		Pearson correlation analysis	1. Greater increase in consequential religiosity (*r* = 0.32, *p* < 0.05) and experiential religiosity (*r* = 0.34, *p* < 0.05) was significantly correlated with greater increase in QOL Lim from baseline to 2-year follow-up 2. Greater increase in religious avoidance coping (*r* = 0.35, *p* < 0.05) and religious coping total scores (*r* = 0.34, *p* < 0.05) was significantly correlated with greater increase in QOL Em from baseline to 2-year follow-up
Trevino et al (2015) USA [32]	Longitudinal study (12 weeks of cardiac rehabilitation)	105	77% men Mean age (SD) = 60.6 years (11.5) 100% White 91% married 72% protestant	First time Myocardial Infarction patients or Post-Coronary Artery bypass Surgical patients		1. SRQC 2. The Religiosity Measure 3. The Religious Coping Activities Scale	QLMI (measured at baseline and 12 weeks)		Spearman Rank correlation analysis of the baseline relationship between R/S and QOL; and between the baseline R/S and QOL changes	1. No significant correlations between the dimensions of R/S and QOL at baseline 2. No significant correlation between R/S measured at baseline and changed value of QOL (12-week QOL Measure minus baseline QOL measure)
Beery et al (2002) USA [33]	Cross-sectional study (final part of a longitudinal study)	58	60% men Mean age = 57 years 90% European American 10% African American Religious Affiliation 62% protestant 29% Catholic 9% other Marital status not reported	Heart Failure No indication of time since diagnosis	Spiritual Well-Being Scale			1. Index of Well-Being 2. HRQOL-SF36 3. MLWHFQ	Correlation Analysis	1. Spiritual well-being was correlated with measures of global QOL (*r* = 0.49, *p* ≤ 0.001), health-related QOL (MCS: *r* = 0.34, *p* ≤ 0.05), and disease-specific QOL (physical symptoms: *r* = − 0.37, *p* ≤ 0.01; emotional symptoms: *r* = − 0.47, *p* ≤ 0.001) 2. Combined spirituality score predicted 24% of the variance in global quality of life
Westlake et al (2002) USA [34]	Cross-sectional study	61	74% men Mean age (SD) = 56.8 years (13.8) 84% White 15% Hispanic 2% Black 72% married No religious affiliation reported	Heart Failure At least 6 months since diagnosis	Spiritual Perspective Scale			HRQOL-SF36	1. Correlation Analysis 2. Multiple linear regression	1. Spirituality was not significantly correlated with the physical (*r* = 0.03, *p* = 0.81) nor mental component of HRQOL (*r* = 0.04, *p* = 0.75) 2. In the multivariable analysis, spirituality was not associated with the physical (*β* = 0.17, *p* = 0.28) nor mental component of HRQOL measure (*β* = 0.08, *p* = 0.54)
Blinderman et al (2008) USA [35]	Cross-sectional data obtained at baseline from longitudinal study	103	71.8% men Mean age (SD) = 67.1 years (12.1) 73% White 13% Black 10% Hispanic 53% married No religious affiliation reported	Congestive heart failure; time since diagnosis not reported	FACIT-Sp-4			MILQ	Correlation Analysis	The FACIT-Sp measure of spirituality was not significantly correlated with MILQ (*r* = 0.16, *p* = 0.11)
Park et al (2008) USA [36]	Longitudinal study (6 months duration)	202 enrolled 163 followed up	60.3% men Mean age = 65.6 years 67% Caucasian 30% African American 3% Latino and other racial categories Marital status not reported Religious affiliation not reported	Left-sided systolic congestive heart failure; diagnosed within one year prior to study enrollment	Religious coping—COPE measure			HRQOL-SF36 (measured at baseline and 6 months of follow-up)	Correlation Analysis	Religious coping measured at baseline was not significantly correlated with the physical (*r* = − 0.11, *p* > 0.05) nor mental (*r* = − 0.05, *p* > 0.05) components of HRQOL measured at 6 months
Bean et al (2009) USA [37]	Cross-sectional study	100	67% men Mean age (SD) = 53 years (14) 49.5% African American 47.4% Caucasian 3.1% Hispanic 51.6% married No religious affiliation reported	Heart failure; time since diagnosis not reported	FACIT-Sp-12			MLWHFQ	Correlation Analysis	1. The meaning/peace subscale of the FACIT-Sp was significantly correlated with QOL (*r* = − 0.43, *p* < 0.01) 2. The faith subscale of the FACIT-Sp was not significantly correlated with QOL (*r* = − 0.06, *p* > 0.05) 3. The total score of the FACIT-Sp was significantly correlated with QOL (*r* = − 0.32, *p* < 0.01)
Bekelman et al (2010) USA [38]	Cross-sectional study	60	63.3% men Median age [IQR] = 75 years [70.81] 11.7% African American 50.9% married No religious affiliation reported	Heart failure; no time since diagnosis reported	1. FACIT-Sp-12 2. IW			KCC Q-QOL	Pearson Correlation Analysis	1. The meaning/peace (*r* = 0.41, *p* = 0.001) and faith (*r* = 0.38, *p* = 0.003) subscales of the FACIT-Sp were significantly correlated with KCCQ-QOL 2. The faith in God subscale of the IW was significantly correlated with KCCQ-QOL (*r* = 0.25, *p* = 0.05) 3. The sense of peace (*r* = 0.21, *p* = 0.10), religious behavior (*r* = 0.09, *p* = 0.52), and compassionate view (*r* = − 0.05, *p* = 0.73) subscales of the IW were not significantly correlated with KCCQ-QOL
Karademas (2010) Greece [39]	Cross-sectional study	135	67.4% men Mean age (SD) = 60.4 years (12.5) 83% married No racial distribution All affiliated to the Orthodox Christian Church	75.5% Myocardial infarction 14.1% Severe angina pectoris 6.7% Arrhythmias 3.7% heart failure; mean time (SD) since diagnosis = 10.7 years (6.4)	1. Intrinsic religiousness 2. Frequency of church service attendance			RAND Health Survey-Physical functioning and Emotional well-being scales	1. Pearson Correlation Analysis 2. Hierarchical regression analyses	1. Intrinsic religiousness was significantly correlated with physical functioning (*r* = 0.26, *p* < 0.01) and emotional well-being (*r* = 0.32, *p* < 0.001) 2. Frequency of church service attendance was significantly correlated with emotional well-being (*r* = 0.20, *p* < 0.05) but not with physical functioning (*r* = 0.06, *p* > 0.05) 3. Intrinsic religiousness was a significant predictor of physical functioning (β = 0.29, *t* = 3.08 *p* < 0.01) and emotional well-being (*β* = 0.28, *t* = 3.05, *p* < 0.001) 4. Frequency of church service attendance was only a significant predictor of emotional well-being (*β* = 0.20, *t* = 2.02, *p* < 0.05)
Hasan et al (2017) Iran [40]	Cross- sectional study	130	47.7% men Mean age (SD) = 59.5 years (12.5) 76.9% Married Religious affiliation: all Muslims No racial distribution provided	Heart Failure At least one year since diagnosis	Islamic religious attitude questionnaire			HRQOL-SF36	1. Pearson Correlation 2. Multiple linear regression	1. Significant correlation between religious attitudes and QOL in the mental (Pearson’s *r* = 0.19, *p* = 0.03) and general health dimensions (Pearson’s *r* = 0.19, *p* = 0.04) 2. No significant correlation between religious attitudes and physical aspect of QOL (Pearson’s *r* = 0.04, *p* = 0.66); nor total QOL scores (Pearson’s *r* = 0.10, *p* = 0.30)
Bang et al (2013) Korea [41]	Cross-sectional study	85	58.5% men Median age (SD) = 26.5 years (5.9) 10.6% Married No religious affiliation nor racial distribution reported	Congenital Heart Disease	Self-reported as religious (Yes/No)			WHOQOL-BREF	Student’s *t* test	Patients who identified as being religious had higher physical health QOL (60.09 ± 12.74 vs 52.64 ± 11.58; *t* = 2.719; *p* value < 0.01) and Environment QOL scores compared to those who did not identify as being religious

*BMMR*/*S* brief multidimensional measure of religion/spirituality, *FACIT-Sp (FACIT-Sp-12; FACIT-Sp-4)* the functional assessment of chronic illness therapy-spiritual well-being (12-item scale; 4-item scale), *HRQOL (SF36; SF12)* Health-Related Quality-of-Life (the 36-item of the Medical Outcomes Study Questionnaire; the 12-item short form of the Medical Outcomes Study Questionnaire), *IW* Ironson–Woods Spirituality/Religiousness Index, *KCCQ-QOL* Quality-of-life Subscale of the self-reported Kansas City Cardiomyopathy Questionnaire, *MCS* Mental Component Score, *MILQ* Multidimensional Index of Life Quality, *MLWHFQ* Minnesota Living with Heart Failure Questionnaire, *QLMI* Quality of life after Acute Myocardial Infarction Questionnaire, *QOL Lim* Quality-of-life Limitations, *QOL Em* Quality-of-life Emotions, *SRCQ* The Spiritual and Religious Concerns Questionnaire, *WHO-BREF* short-version of the World Health Organization QoL assessment

### Measures of R/S

The dimensions of R/S assessed in the identified studies included religious attitudes [40], religious, existential, and spiritual well-being [33, 37, 38], religious support [28], spiritual perspectives [34], strength and comfort from religion [35], religious coping [28, 31, 32, 36], church service attendance [39], intrinsic religiousness [39], religious identification and religious struggle [27, 28], spiritual desires and constraints [30], spiritual and religious concerns [32], belief in the afterlife [28], forgiveness [28], and daily spiritual experience [27, 28, 30]. A variety of instruments were used to assess R/S (Table 2) ranging from a simple validated one-item scale [39] to a more complex 29-item scale [42]. Three instruments that assessed R/S were used in more than one study: the 12-item Functional Assessment of Chronic Illness Therapy (FACIT-Sp-12), a validated self-reported measure of overall spiritual well-being that assesses “Meaning/Peace” and “Faith” [43], the Brief Multidimensional Measure of Religion/Spirituality (BMMR/S) [44], and the Religious strain scale [45]. Table 2 provides a description of the R/S instruments and scoring systems used in the included studies.

**Table 2:** Religiosity and / or spirituality measures used in the included studies

Religiosity and/or spirituality measure [reference]	Number of items	Instrument description	Scoring system	Studies that used measure in this review
Spiritual desires, constraints, and needs questionnaire [30]	3	Instrument developed for this specific study based on prior qualitative study findings on spiritual needs. Items were “‘Do you want your doctor and other healthcare providers to attend to your spiritual needs?,’ ‘How much do you feel limited or constrained in discussing your spiritual issues with your doctor and other health care providers?,’ and ‘How well are your spiritual needs getting met right now?.’” No psychometric properties of the scale or validation procedure were reported	Responses for each item ranged from 1 (not at all) to 4 (very much/a great deal)	[30]
Church service attendance [39]	1	Single item assessing the frequency of church service attendance in the previous 6 months	Item response is scored using a five-point Likert-type scale ranging from 1 (least frequency of attendance) to 5 (most frequent service attendance)	[39]
Islamic religious attitude questionnaire [40]	25	A self-report scale with 6 dimensions on learning and reading the Quran; Knowledge of God and faith in God; belief in afterlife; attitude to Islamic religious rituals; positive attributes; devotion to religious worship; and praying. The instrument was developed for the purpose of the study [11]. Psychometric validation of the instrument was conducted with test–retest correlation coefficient of 0.86 and internal consistency Cronbach’s *α* = 0.89	4-point Likert Scale with response items ranging from 1 = strongly disagree to 4 = strongly agree	[40]
The Religious Coping Activities Scale [42]	29	A validated instrument which assesses the degree to which people use religion to cope with stressful life events. Six types of religious coping are assessed: spiritually based activities (12 items), good deeds (6 items), discontentment (3 items), interpersonal religious support (2 items), pleading and bargaining with a Supreme Being (3 items), and religious avoidance (3 items)	A 4-point Likert scale is used to assess how participants rely on each religious coping strategy. Higher scores imply greater reliance on religion for coping. Subscale and total scores are derived from the mean of the individual items	[31, 32]
Functional Assessment of Chronic Illness Therapy FACIT-Sp-12 [43]	12	A validated self-report measure of overall spiritual well-being. Two subscales are assessed: “Meaning/Peace” (8 items) and Faith (4 items). The meaning/peace subscale assesses one’s sense of meaning, peace, harmony, and life’s purpose. The faith subscale measures the relationship between faith, spiritual beliefs, and illness, and seeking solace in one’s faith	The response to each item ranges from 0 (not at all) to 4 (very much). A composite score ranging from 0 to 48 is derived from the subscales with higher scores indicating greater spiritual well-being	[37, 38]
Religious identification [44]	1	A validated measure of the extent to which an individual considered themselves religious	Scored from 0 (not at all) to 4 (extremely). Dichotomized in the study as low and high	[27]
Religious comfort—Daily spiritual experience scale [44]	3	Religious comfort assessed from the Daily spiritual experience scale. Respondents rate how they feel about the presence of God, derived comfort or strength in their religion or spirituality, and experienced God’s love directly or via others	Responses range from 0 (never or almost never to 6 (many times a day), with higher scores reflecting greater religious comfort	[27]
Brief Multidimensional Measure of Religion/Spirituality (BMMR/S) [44]	23	The following dimensions of religiousness/spirituality is assessed with the BMMMR/S: Forgiveness (3 items), daily spiritual experiences (8 items), belief in life after death (1 item), religious identity (1 item), religious support (2 items), public religious practices (2 items), and positive religious/spiritual coping (4 items)	Each dimension is scored separately: Forgiveness (1–4), daily spiritual experience (1—never to 8—many times a day), belief in life after death (0—no, 1—undecided, 2—yes), religious identity (0—not considered a religious person to 4—extremely religious), religious support (1—none to 4—a great deal), public religious practices (1—never to 8—several times a week), positive R/S coping (1—not at all to 4—a great deal)	[28, 30]
Religious struggle—Religious strain scale [45]	6	Instrument derived from the brief version of the religious strain scale. Respondents rate their agreement with the items on their feeling of anger or alienation from God	Responses range from 0 (not at all) to 10 (extremely). Summed scores range from 0 to 60, with higher scores implying greater religious struggle	[27, 28]
Spiritual Well-Being Scale [46]	20	A validated 10-item subscales assessing religious well-being (RWB) and existential well-being (EWB), respectively. Items on the RWB make direct reference to God while items on the EWB measure a sense of purpose or meaning to life with direct reference to God	6-point Likert scale where higher numbers indicate greater endorsement of the statement. Negative items are reversely scored. The 10 items are scored from 10 to 60 and the scores from the two subscales can be added to derive an overall spiritual well-being score ranging from 20 to 120 with higher scores indicating better spiritual well-being	[33]
The Spiritual Perspective Scale [47]	10	A validated measure of spirituality with adequate psychometric properties. The items measure the extent to which spirituality permeates one’s life, one’s engagement in spiritually related interactions, perceived spiritual perspectives, and an individuals’ practice and belief system	There are 5 response options scored from 1 (not at all/strongly disagree) to 6 (about once a day/strongly agree). The total score ranges from 10 to 60, higher scores indicate greater spiritual perspective and higher levels of self-transcendence	[34]
Functional Assessment of Chronic Illness Therapy FACIT-Sp-4 [48]	4	Derived from the FACIT-Sp-12. Measures the extent of strength and comfort derived from one’s faith	Scores range from 0 to 4, with higher scores indicating greater spirituality	[35]
Religious coping—COPE measure [49]	4	The COPE measure is a validated 60-item instrument with 15 subscales that measures how individuals cope with stressful life situations. The Religious subscale (4 items) assesses how people turn to religion by seeking God’s help, putting their trust in God, finding comfort in their religion, and praying more than usual during stressful periods	The response to each item is scored from 1 (I usually do not do this at all)–4 (I usually do this a lot), indicating the frequency with which an individual carries out religious coping. Subscales are assessed individually with scores ranging from 4 to 16. Higher scores imply greater religious coping	[36]
Ironson–Woods Spirituality/Religiousness Index (IW) [50]	25	A validated self-report instrument that measures spirituality in two dimensions: traditional religiousness and private spirituality. Four subscales assess an individuals’ “sense of peace” (9 items), “faith in God” (6 items), “religious behavior” (5 items), and “compassion view of others” (5 items)	Responses indicate how strongly one agrees with each item with scores from 1 (strongly disagree) to 5 (strongly agree)	[38]
Intrinsic religiousness [51]	9	The Intrinsic religiousness subscale is derived from the Religious Orientation Scale	Responses are scored using a five-point Likert-type scale, with lower scores indicating higher intrinsic religiousness	[39]
The Religiosity Measure [52]	8	A validated instrument which assesses the impact of religion on an individual’s daily life. Comprises four subscales with two items each: ritual religiosity, consequential religiosity, ideological religiosity, and experiential religiosity. Ritual religiosity assesses the frequency of attendance in religious services, and the practice of meditation or prayer. Consequential religiosity measures the extent to which religion affects respondent’s decision and daily life. Ideological religiosity assesses belief in a Supreme Being and life after death. Experiential religiosity assesses the respondent’s religious devotion and comfort from religion	Each item is scored on a 5-point Likert scale from 0 (least religiosity) to 4 (greatest religiosity) except the item on religious service attendance that is scored from 1 to 4 with increasing frequency of service attendance. Each subscale has a maximum score of 8 and the overall score for the religiosity measure is 32	[31, 32]
The Spiritual and Religious Concerns Questionnaire (SRQC) [53]	11	A validated instrument which assesses the strength of spiritual beliefs (7 items) and religious practices (4 items). Originally designed to assess spiritual concerns in adolescents who were hospitalized. Adapted for use in adult population to assess spiritual concerns broadly and in keeping with the respondent’s illness	Each response is scored from 1 (least spiritual/religious) to 9 (most spiritual/religious). The overall score is derived from the mean of the 11 items	[32]

### QOL outcomes

In contrast to the different measures of R/S, the QOL outcomes were more homogenous across studies (Table 3). Commonly reported outcomes were global QOL, mental or physical HRQOL, disease-related QOL, and dimensions of functional, emotional, or social well-being. Global QOL was assessed in three studies [33, 35, 41] with a different instrument used in each study: The Index of Well-Being [54], Short-version of The World Health Organization QoL assessment (WHOQOL-BREF) [55], and the Multidimensional Index of Life Quality (MILQ) [56]. Nine studies evaluated patients’ HRQOL using three instruments: The 36-item Medical Outcomes Study Questionnaire (SF-36) [57] was used in four studies [33, 34, 36, 40], the 12-item Short Form of the Medical Outcomes Study Questionnaire (SF-12) [58] was used in four studies [27–30], and the RAND 36-item Health Survey [59], a validated instrument adapted from the SF-36 that uses a simpler scoring system, was utilized in one study [39]. Five studies assessed disease-specific QOL with three instruments: The Minnesota Living with Heart Failure (MLHF) Questionnaire [60] in two studies [33, 37], the Quality of Life after Acute Myocardial Infarction (QLMI) [61] in two studies [31, 32], and the Kansas City Cardiomyopathy Questionnaire (KCCQ)-QOL subscale [62] in a single study [38].

**Table 3:** Quality-of-life (QOL) measures used in the included studies

Quality-of-life measurement [reference]	Number of items	Instrument description	Scoring system	Studies which used measure in this review
Global quality-of-life measure				
Index of Well-Being [54]	9	A validated measure of well-being. Comprises 8 specific items about the individual’s perception of their life. The final item measures their overall satisfaction with life. Each item is rated on a 7-point rating system with a positive aspect on one end and a negative aspect at the other end	The first 8 items have a mean weighted at 1.0 which is added to the score for the last item weighted at 1.1. The total possible scores range from 2.1 (lowest life satisfaction) to 14.7 (highest life satisfaction)	[33]
Short-version of The World Health Organization QoL assessment (WHOQOL-BREF) [55]	26	The WHOQOL-BREF is a shortened version of the WHOQOL-100 which provides a detailed assessment of QOL but may be too lengthy for practical use. 24 items derived from the WHOQOL-100 are used to assess four domains including an individuals’ perception of their physical health (7 items), psychological health (4 items), social relationships (3 items), and their environment (8 items). Two additional questions assess the overall QOL and general health	The items are used to derive a mean score for their respective domain. The additional items are rated on a 5-point Likert scale (1- least score to 5-highest score). The mean score for each domain is transformed in two stages. First, the mean score is multiplied by 4 to derive a score ranging from 4 to 20 which is comparable with the WHOQOL-100 score. Second, the domain scores are converted to a 0-100 scale with higher scores implying better QOL	[41]
Multidimensional Index of Life Quality (MILQ) [56]	35	The MILQ is a validated, patient self-reported instrument that assesses 9 domains, namely, physical, cognitive, and social functioning; physical and mental health; productivity, financial status, intimacy, and relationship with health professionals	Each item is scored on a 7-point Likert scale from 1 (very dissatisfied) to 7 (very satisfied). All subscores of the MILQ are scored with a range from 4 to 28. The composite score ranges from 8 to 24, and is derived as a weighted sum of an individuals’ global QOL	[35]
Health-related quality-of-life measure				
The 36-item Medical Outcomes Study Questionnaire (SF-36) [57]	36	A standardized measure of generic health-related QOL with close-ended structured questions. There are 8 dimensions, 4 of which comprise the Physical Component Score (PCS) including measures of limitation in physical functioning, physical health problems with resultant role limitations, bodily pain, and general health perceptions. The other 4 dimensions which comprise the Mental Component Score (MCS) include vitality, social functioning, emotional problems with resultant role limitations, and general mental health	Each respective dimension transformed into 0–100 scale. Higher scores indicate better QOL	[33, 34, 36, 40]
The 12-item Short Form of the Medical Outcomes Study Questionnaire (SF-12) [58]	12	Valid measure which assesses two dimensions of QOL: physical health component (measures of general health, pain assessment, fatigue, physical functioning, and interference of role performance due to physical health limitations), and a mental health component (measures of emotional well-being, vitality, social functioning, and role interference due to emotional health limitations)	Items are measured on different scales including ‘yes’/’no,’ ‘not at all’/‘very much.’ A mean score is generated for each component ranging from 0 to 100. The subscales are normed on the general adult US population with a mean (SD) of 50 (10). Higher scores indicates better HRQOL	[27–30]
RAND 36-item health survey [59]	36	The items in the RAND Health survey were adapted from the 36-item Medical Outcomes Study Questionnaire (SF-36), although having a simpler scoring system. Eight domains are assessed, namely, bodily pain (2 items), energy/fatigue (4 items), physical functioning (10 items), role limitations from physical health problems (4 items), emotional well-being (5 items), role limitations due to emotional problems (3 items), social functioning (2 items), and general health perceptions (5 items). A single item measures perceived change in health	Each item is scored from 0 to 100 (higher scores indicate more favorable health). The items in each domain are averaged to create 8-scale scores	[39]
Disease-specific quality-of-life measure				
Minnesota Living with Heart Failure Questionnaire (MLHF) [60]	21	A validated Likert-type instrument created for assessing health-related QOL among patients diagnosed with heart failure. Measures the effect of heart failure on physical and emotional dimensions of life. The physical items include symptoms such as fatigue, swelling, shortness of breath, role functioning with difficulty performing work or social activities. The emotional items assess worry, depression, and losing self-control	Each item is rated from 0 (did not) to 5 (very much prevented me from living as I wanted). Higher scores on the physical and emotional subscales imply lower quality of life	[33, 37]
Quality of Life after Acute Myocardial Infarction (QLMI) [61]	25	Validated and reliable disease-specific instrument which consists of two subscales: The Limitations (QOL Lim) subscale which assesses the frequency of physical symptoms and how much it interferes with daily life, and the Emotional subscale (QOL Em) which assesses patients’ self-esteem, emotional well-being, and ability to manage their illness	The scores range from 1 (all of the time) to 7 (none of the time) with a mean response to all 25 items. Higher scores indicate better QOL	[31, 32]
Kansas City Cardiomyopathy Questionnaire (KCCQ)-QOL subscale [62]	3	The QOL subscale of the self-reported KCCQ is a validated measure which assesses how heart failure impacts patient’s overall QOL. Two items address QOL, while the third item assesses depression	Scored from 0 to 100 with a higher score indicating better QOL	[38]

### Statistical analysis

Eight studies conducted a correlational analysis only [29, 31–33, 35–38], three studies conducted both correlation and hierarchical regression analyses [27, 28, 39], two studies conducted both correlation and multiple linear regression analyses [34, 40], one study utilized only multiple regression analysis [30], and another study used *t* tests to assess between group differences [41]. Correlation analysis was conducted between R/S and QOL measured at a single time point in cross-sectional studies [33–35, 37–40]. In studies using a longitudinal design, researchers examined the association between R/S measured at baseline and QOL during the course of follow-up [27–29, 31, 32, 36]. Socio-demographic variables commonly adjusted for in the regression analyses included age, gender, race, marital status, and education.

### Association between R/S and QOL

The association between R/S and QOL differed according to the dimension of R/S and QOL domain assessed. We have summarized the principal study findings based on the association between R/S and QOL domain examined (global QOL, HRQOL, and disease-specific QOL), and according to the type of CVD. Results from included studies are detailed in Table 1.

### Association between R/S and QOL in patients with heart failure

Eleven studies examined the association between R/S and QOL in patients with heart failure. Four publications used longitudinal data [27–29, 36], and seven used a cross-sectional design [30, 33–35, 37, 38, 40]. A significant positive association between R/S and QOL was reported in six of the eleven studies [28–30, 33, 38, 40]. The association between R/S and QOL domains in patients with heart failure is as follows:

R/S and Global QOL: Spiritual well-being was positively correlated with global QOL measures (r = 0.49, *p* ≤ 0.001) [33], while spirituality, as assessed with the FACIT-Sp measure, was not significantly related to global QOL [35].

R/S and HRQOL: Higher daily spiritual experience and having one’s spiritual needs met were positively associated with higher physical well-being [28, 30]. Attending to one’s spiritual needs, spiritual well-being, and a more religious attitude were positively associated with better mental or emotional well-being [30, 33, 40]. However, belief in the afterlife at 1 month was negatively associated with mental HRQOL at 3 months [28], while spiritual constraint was associated with poorer physical QOL [30]. Spirituality was not associated with the physical or mental components of HRQOL [34]. Neither religious struggle nor religious coping were significantly associated with the mental or physical components of HRQOL at study baseline and during a subsequent follow-up evaluation [27, 36].

R/S and disease-specific QOL: A cross-sectional study assessed patient’s spirituality with the FACIT-Sp and Ironson–Woods Spirituality/Religiousness Index (IW) [38]; the meaning/peace and faith subscales of the FACIT-Sp, and the faith in God subscale of the IW were positively correlated with QOL, as assessed with the KCCQ. In contrast, the sense of peace, religious behavior, and compassionate view subscales of the IW were not significantly associated with KCCQ-QOL. Another study found no significant association between the meaning/peace and faith subscales of the FACIT-Sp and QOL as assessed with the MLHF questionnaire [37]. Lower spiritual well-being was negatively associated with poorer physical and emotional symptoms [33].

### Association between R/S and QOL in patients with acute myocardial infarction (AMI)

Two longitudinal studies examined the association between R/S and disease-specific QOL in patients with AMI [31, 32]. The findings from a study of 105 patients with a first time AMI [31], showed that higher consequential religiosity, experiential religiosity, and religious avoidance coping were significantly associated with increases in QOL from baseline to the 2-year follow-up. In this cohort, no significant association was found between the dimensions of R/S and QOL at baseline, and the baseline measure of R/S was not associated with changes in QOL after 12-weeks of cardiac rehabilitation [32].

### Association between R/S and QOL in congenital heart disease

One cross-sectional study examined the association between R/S and global QOL in patients with congenital heart disease [41]. Those who identified as being religious had higher physical and environmental QOL scores (60.1 vs 52.6; *p* value < 0.01) compared with those who did not identify as being religious.

### Association between R/S and QOL in a study with multiple CVD diagnoses

A cross-sectional study that enrolled patients (*n* = 135) with varying CVD diagnoses (75.5% myocardial infarction, 14.1% severe angina pectoris, 6.7% arrhythmias, and 3.7% heart failure) examined the association between R/S and HRQOL [39]. Intrinsic religiousness was positively associated with higher emotional and physical well-being, and a higher frequency of church attendance was positively associated with better mental or emotional well-being.

### Study quality assessment

The quality scores of the included studies ranged from 73.7 to 94.7%. All studies clearly reported their objective(s), described study participant characteristics, and their key exposure and outcome variables. With respect to internal validity, only a few studies sufficiently adjusted for potential confounders in the form of a multivariable regression analysis [27, 28, 34, 39, 40]. Five longitudinal studies [27–29, 31, 36] adequately reported the number of participants recruited, those lost to follow-up, and reasons for attrition. Most studies addressed the representativeness of their study sample and the generalizability of their findings to the population from which the study subjects were selected.

## Discussion

In this systematic review, we found evidence for an association between R/S and QOL among patients with CVD. This association varied depending on the dimension of R/S and QOL domain assessed. Ten of the fifteen studies identified in this review reported a significant positive association between R/S and QOL, and approximately half of the studies reported negative or null associations. The majority of included studies were conducted among patients with heart failure.

Prior studies have posited a variety of mechanisms by which R/S influences QOL in patients with chronic conditions. Religiousness has been shown to enhance self-esteem, generate positive emotions, and promote positive self-care practices by encouraging individuals to refrain from unhealthy lifestyle practices, which in turn fosters well-being [63–65]. R/S may favorably influence an individual’s QOL by fostering a deeper sense of meaning when faced with life-threatening or chronic debilitating conditions [66]. Our findings suggest that R/S is associated with QOL, as intrinsic religiousness, spiritual well-being, and attending to one’s spiritual needs were related to better physical, mental, and emotional functioning. On the other hand, spiritual constraint and lower spiritual well-being were associated with poorer physical and emotional well-being.

### R/S and QOL

We observed considerable heterogeneity in the R/S measures utilized, reflective of the varying dimensions of R/S assessed in research and the general lack of consensus in defining R/S [67]. Most of the included studies utilized already existing validated scales, whereas one recent study designed their R/S questionnaire for purposes of assessing religious attitudes [40]. This latter study provided a detailed description of their instrument validation process and had a high-quality rating in our methodological assessment.

Upwards of sixteen dimensions of R/S were assessed across studies with religious coping being the most commonly assessed aspect. In patients diagnosed with an initial AMI or after coronary artery bypass surgery, higher religious coping was associated with better emotional QOL over a 2-year follow-up period. In contrast, no association was observed between religious coping and physical/mental well-being in patients living with heart failure at 6 months of follow-up. These findings reflect how R/S may differentially influence QOL depending on the domain assessed, patient’s clinical diagnosis, and the duration of follow-up in assessing the impact of one’s R/S on their QOL since shorter follow-up periods may not sufficiently allow for R/S to influence health outcomes. Furthermore, reverse causation and residual confounding may explain these differences observed in the various studies included in this review. In a study [28] that examined seven dimensions of R/S (forgiveness, daily spiritual experiences, belief in afterlife, religious identity, religious support, public practices, and positive RS coping), only moderate correlations were found between the dimensions suggesting that they each represent a unique aspect of one’s religious/spiritual experience, and that each R/S dimension may have a distinct role in the relationship between R/S and patient’s QOL domains.

We observed considerably greater uniformity in the QOL outcome measures examined in this review, with the three major domains of global, health-related, and disease-specific QOL assessed.

## Summary of the Literature

A majority of studies included in this review [*n* = 11] were conducted between 2010 and 2017, indicative of an increasing awareness of the relationship between R/S and patient’s QOL. Most of the identified studies [*n* = 11] enrolled patients with heart failure, which may be attributable to the worldwide rise in the magnitude of heart failure and its considerable morbidity and mortality, and impact on patient’s QOL due to its physical and emotional symptoms [68, 69]. However, future research is needed among patients with varying manifestations of underlying CVD, including acute and chronic forms of heart disease, which may have a considerable impact on patient’s QOL.

Most of the included studies [*n* = 12] were conducted in the US, and the study participants were predominantly non-Hispanic Whites and middle-older aged persons, which limits the generalizability of the study findings to ethnic minority groups and younger individuals. There was an overrepresentation of studies with small sample sizes, short follow-up duration, or the use of a cross-sectional design, which limits the conclusiveness of our review. Results from the methodological quality assessment revealed that included studies had moderate- to high-quality ratings, which lends some credence to the reliability of our findings.

### Strengths and limitations of the current systematic review

To our knowledge, this is the first systematic review to examine the association between R/S and QOL in patients with CVD. From a self-evaluation of our review using the AMSTAR tool for assessing systematic review quality [70], we obtained a score of 10 out of a maximum of 11 points. The one point not credited to this review was due to our inability to investigate possible publication bias with a funnel plot as we would have required a uniform measure of effect, which was impossible due to heterogeneity in the assessment of R/S across the included studies.

Several limitations of our review exist. First, we excluded non-English articles, likely leading to publication bias. Our initial search of the electronic databases did not exclude studies based on publication language; however, only four studies in foreign languages were identified. Second, we suggest caution in interpreting the synthesized results from this review, as causal inferences on the association between R/S and QOL cannot be made from observational studies which are susceptible to potential confounding by unmeasured or inadequately measured variables, and cross-sectional studies do not account for temporality. Most included studies were conducted among patients with heart failure, which may have limited the generalizability of our findings. Lastly, most identified studies were conducted in the US, which may not adequately capture R/S and cultural impact on QOL from a global perspective.

### Research and clinical implications

Future research should be conducted in patients with different CVD conditions to better understand how R/S may influence their QOL. Longitudinal studies in larger patient samples are needed to better understand how R/S may affect QOL over varying follow-up periods, as it is unclear whether any associations observed over the short-term persist on a longer-term basis. Future studies should evaluate how patients may turn to or away from R/S in periods of illness and stress, how this might influence their QOL, and identify those in need of clerical intervention for a more holistic approach in patient management. In addition, there is a need for uniformity in assessing R/S to ensure more reliable and comparable results across studies. Furthermore, advanced analytic techniques, such as propensity scoring and instrumental variables to address confounding in observational studies should be explored.

The findings from this review reveal that certain dimensions of R/S are likely associated with patient’s QOL. Healthcare providers need to consider the influence of R/S on patient’s QOL, as this may also influence patient engagement with their treatment and long-term outcomes.

## Electronic supplementary material

Below is the link to the electronic supplementary material.

Supplementary material 1 (DOCX 21 KB)
