# Supplementary material for: Association of religiosity and spirituality with quality of life in patients with cardiovascular disease: a systematic review
Source: Qual Life Res. 2018 Jun 11;27(11):2777–97. doi: 10.1007/s11136-018-1906-4 (PMC6196107; doi:10.1007/s11136-018-1906-4)
Supplement: Supplementary file 1 — Supplementary material 1 (DOCX 21 KB) [file 11136_2018_1906_MOESM1_ESM.docx]

**Electronic Supplementary Material**

**Article title:** Association of Religiosity and Spirituality with Quality of Life in Patients with Cardiovascular Disease: A systematic review

**Journal name:** Quality of Life Research Journal

**Authors:** Hawa O. Abu, MD, MPH Christine Ulbricht, PhD Eric Ding, MS Elena Salmoirago-Blotcher MD, PhD Robert J. Goldberg, PhD Catarina I. Kiefe, PhD, MD

**Corresponding author:**

Hawa Abu, MBBS, MPH

Department of Quantitative Health Sciences

University of Massachusetts Medical School

368 Plantation Street, Worcester, MA 01605

E-mail: Hawa.Abu@umassmed.edu

**Supplement 1_Search Algorithm Used in the Electronic Databases**

Period search was conducted: September 15 2017 - October 20 2017

No restriction on publication date or publication language

PubMed Search

((((((("Religion"[Mesh]) OR Religion) OR Religiousness) OR Religiosity OR “Religious Coping”)) OR ((("Spirituality"[Mesh]) OR Spirituality) OR Spiritual)) AND (((("Quality of Life" [Mesh]) OR “Health Related Quality of Life” OR “Well-being”) OR QOL) OR “Quality of Life”)) AND (((((("Cardiovascular Diseases"[Mesh]) OR “Cardiovascular disease”) OR “Acute Myocardial Infarction”) OR Acute Coronary Syndrome) OR Congenital Heart Disease) OR “Rheumatic Heart Disease” OR “Heart Failure” OR “Cardiac Surgery”)

SCOPUS (Elsevier) Search

TITLE-ABS-KEY (religion OR religiousness OR religiosity OR “Religious Coping” OR spirituality OR spiritual) AND TITLE-ABS-KEY ("Quality of Life" OR "Health Related Quality of Life" OR QOL OR “Well-being”) AND TITLE-ABS-KEY ("Cardiovascular Disease" OR "Acute Myocardial Infarction" OR "Acute Coronary Syndrome" OR "Congenital Heart Disease" OR "Rheumatic Heart Disease" OR “Heart Failure” OR “Cardiac Surgery”)

PsycInfo Search

religion OR religiousness OR religiosity OR “religious coping” OR spirituality OR spiritual AND "Quality of Life" OR "Health Related Quality of Life" OR QOL OR “well-being” AND "Cardiovascular Disease" OR "Acute Myocardial Infarction" OR "Acute Coronary Syndrome" OR "Congenital Heart Disease" OR "Rheumatic Heart Disease" OR “Heart failure” OR “Cardiac surgery”

CINAHL Search

(religion OR religiousness OR religiosity OR “religious coping” OR spirituality OR spiritual) AND ("Quality of Life" OR "Health Related Quality of Life" OR QOL OR “well-being”) AND ("Cardiovascular Disease" OR "Acute Myocardial Infarction" OR "Acute Coronary Syndrome" OR "Congenital Heart Disease" OR "Rheumatic Heart Disease" OR “Heart failure” OR “Cardiac surgery”)

**Supplement 2_Modified Down and Black Checklist for Study Quality Assessment**

| **Criteria** | **Applicable Study Design** | Park et al, 2011 [27] | Park et al, 2014 [28] | Sacco et al, 2014 [29] | Park & Sacco, 2017 [30] | Trevino et al, 2014 [31] | Trevino et al, 2015 [32] | Beery et al, 2002 [33] | Westlake et al, 2002 [34] | Blinderman et al, 2008 [35] | Park et al, 2008 [36] | Bean et al, 2009 [37] | Bekelman et al, 2010 [38] | Karademas, 2010 [39] | Hasan et al, 2017 [40] | Bang et al, 2013 [41] |
| --- | --- | --- | --- | --- | --- | --- | --- | --- | --- | --- | --- | --- | --- | --- | --- | --- |
| ***1. Reporting***  Is the hypothesis/aim/objective of the study clearly described?  (Yes = 1, No=0) | **All studies** | 1 | 1 | 1 | 1 | 1 | 1 | 1 | 1 | 1 | 1 | 1 | 1 | 1 | 1 | 1 |
| ***2. Are the main outcomes to be measured clearly described in the Introduction or Methods section?***  If the main outcomes are first mentioned in the Results section, the question should be answered no. (Yes = 1, No=0) | **All studies** | 1 | 1 | 1 | 1 | 1 | 1 | 1 | 1 | 1 | 1 | 1 | 1 | 1 | 1 | 1 |
| ***3. Are the characteristics of the patients included in the study clearly described?***  In cohort studies and trials, inclusion and/or exclusion criteria should be given. In case-control studies, a case-definition and the source for controls should be given. (Yes = 1, No =0) | **All studies** | 1 | 1 | 1 | 1 | 1 | 1 | 1 | 1 | 1 | 1 | 1 | 1 | 1 | 1 | 1 |
| ***4. Are the distributions of principal confounders in each group of subjects to be compared clearly described?***  A list of principal confounders is provided. (Yes=2, Partially=1, No=0) | **All studies** | 2 | 2 | 2 | 2 | 2 | 1 | 1 | 1 | 2 | 2 | 2 | 2 | 2 | 2 | 2 |
| ***5. Are the main findings of the study clearly described?***  Simple outcome data (including denominators and numerators) should be reported for all major findings so that the reader can check the major analyses and conclusions. (This question does not cover statistical tests which are considered elsewhere).  (Yes= 1, No=0) | **All studies** | 1 | 1 | 1 | 1 | 1 | 1 | 1 | 1 | 1 | 1 | 1 | 1 | 1 | 1 | 1 |
| ***6. Does the study provide estimates of the random variability in the data for the main outcomes?***  In non-normally distributed data the inter-quartile range of results should be reported. In normally distributed data the standard error, standard deviation or confidence intervals should be reported. If the distribution of the data is not described, it must be assumed that the estimates used were appropriate and the question should be answered yes. (Yes=1, No=0) | **All studies** | 1 | 1 | 1 | 1 | 1 | 1 | 1 | 1 | 1 | 1 | 0 | 1 | 1 | 1 | 1 |
| ***7. Have the characteristics of patients lost to follow-up been described?***  This should be answered yes where there were no losses to follow-up or where losses to follow-up were so small that findings would be unaffected by their inclusion. This should be answered no - where a study does not report the number of patients lost to follow-up. (Yes=1, No=0) | **Longitudinal studies** | 1 | 1 | 1 |  | 1 | 0 |  |  |  | 1 |  |  |  |  |  |
| ***8. Have actual probability values been reported?***  E.g. 0.035 rather than <0.05 for the main outcomes except where the probability value is less than 0.001? (Yes=1, No=0) | **All studies** | 0 | 0 | 0 | 0 | 1 | 1 | 1 | 1 | 1 | 0 | 0 | 1 | 0 | 1 | 1 |
| **External validity: All the subsequent criteria attempt to address the representativeness of the findings of the study and whether they may be generalized to the population from which the study subjects were derived.** |  |  |  |  |  |  |  |  |  |  |  |  |  |  |  |  |
| ***9. Were the subjects asked to participate in the study representative of the entire population from which they were recruited?***  The study must identify the source population for patients and describe how the patients were selected. Patients would be representative if they comprised the entire source population, an unselected sample of consecutive patients, or a random sample. Random sampling is only feasible where a list of all members of the relevant population exists. Where a study does not report the proportion of the source population from which the patients are derived, the question should be answered as unable to determine. (1 = Yes, 0 = No / Indeterminate) | **All studies** | 1 | 1 | 1 | 1 | 1 | 1 | 1 | 1 | 1 | 1 | 1 | 1 | 1 | 1 | 1 |
| ***10. Were those subjects who were prepared to participate representative of the entire population from which they were recruited?***  The proportion of those asked who agreed should be stated. Validation that the sample was representative would include demonstrating that the distribution of the main confounding factors was the same in the study sample and the source population. (1 = Yes, 0 = No / Indeterminate) | **All studies** | 0 | 0 | 0 | 0 | 0 | 0 | 1 | 0 | 0 | 0 | 0 | 0 | 1 | 0 | 1 |
| **Internal validity – bias** |  |  |  |  |  |  |  |  |  |  |  |  |  |  |  |  |
| ***11. If any of the results of the study were based on “data dredging”, was this made clear?***  Any analyses that had not been planned at the outset of the study should be clearly indicated. If no retrospective unplanned subgroup analyses were reported, then answer yes. (1 = Yes, 0 = No / Indeterminate) | **All studies** | 1 | 0 | 1 | 1 | 1 | 1 | 1 | 1 | 1 | 1 | 1 | 1 | 1 | 1 | 1 |
| ***12. In trials and cohort studies, do the analyses adjust for different lengths of follow-up of patients, or in case-control studies, is the time period between the intervention and outcome the same for cases and controls?***  Where follow-up was the same for all study patients the answer should yes. If different lengths of follow-up were adjusted for by, for example, survival analysis the answer should be yes. Studies where differences in follow-up are ignored should be answered no.  (1 = Yes, 0 = No / Indeterminate) | **Longitudinal Studies only** | 1 | 1 | 1 |  | 1 | 1 |  |  |  | 1 |  |  |  |  |  |
| ***13. Were the statistical tests used to assess the main outcomes appropriate?***  The statistical techniques used must be appropriate to the data. For example, nonparametric methods should be used for small sample sizes. Where little statistical analysis has been undertaken but where there is no evidence of bias, the question should be answered yes. If the distribution of the data (normal or not) is not described it must be assumed that the estimates used were appropriate and the question should be answered yes.  (1 = Yes, 0 = No / Indeterminate) | **All studies** | 1 | 1 | 1 | 1 | 1 | 1 | 1 | 1 | 1 | 1 | 1 | 1 | 1 | 1 | 1 |
| ***14. Were the main outcome measures used accurate (valid and reliable)?***  For studies where the outcome measures are clearly described, the question should be answered yes. For studies which refer to other work or that demonstrates the outcome measures are accurate; the question should be answered as yes. (1 = Yes, 0 = No / Indeterminate) | **All Studies** | 1 | 1 | 1 | 1 | 1 | 1 | 1 | 1 | 1 | 1 | 1 | 1 | 1 | 1 | 1 |
| **Internal Validity –confounding (selection bias)** |  |  |  |  |  |  |  |  |  |  |  |  |  |  |  |  |
| ***15. Were the patients in different intervention groups (trials and cohort studies) or were the cases and controls (case-control studies) recruited from the same population?***  For example, patients for all comparison groups should be selected from the same hospital. The question should be answered unable to determine for cohort and case-control studies where there is no information concerning the source of patients included in the study.  (1 = Yes, 0 = No / Indeterminate) | **Longitudinal studies** | 1 | 1 | 1 |  | 1 | 1 |  |  |  | 1 |  |  |  |  |  |
| ***16. Were study subjects in different intervention groups (trials and cohort studies) or were the cases and controls (case-control studies) recruited over the same period of time?***  For a study which does not specify the time period over which patients were recruited, the question should be answered as unable to determine  (1 = Yes, 0 = No / Indeterminate) | **Longitudinal studies** | 1 | 1 | 1 |  | 1 | 1 |  |  |  | 1 |  |  |  |  |  |
| ***17. Was there adequate adjustment for confounding in the analyses from which the main findings were drawn?***  This question should be answered no for trials if: the main conclusions of the study were based on analyses of treatment rather than intention to treat; the distribution of known confounders in the different treatment groups was not described; or the distribution of known confounders differed between the treatment groups but was not taken into account in the analyses. In nonrandomized studies if the effect of the main confounders was not investigated or confounding was demonstrated but no adjustment was made in the final analyses the question should be answered as no. (1 = Yes, 0 = No / Indeterminate) | **All studies** | 1 | 1 | 0 | 1 | 1 | 0 | 0 | 1 | 1 | 1 | 1 | 0 | 1 | 1 | 0 |
| ***18. Were losses of patients to follow-up taken into account?***  If the numbers of patients lost to follow-up are not reported, the question should be answered as unable to determine. If the  proportion lost to follow-up was too small to affect the main findings, the question should be answered yes. (1 = Yes, 0 = No / Indeterminate) | **Longitudinal Studies** | 1 | 1 | 1 |  | 1 | 0 |  |  |  | 1 |  |  |  |  |  |
| **Total score earned** |  | 17 | 16 | 16 | 12 | 18 | 14 | 12 | 13 | 13 | 17 | 11 | 12 | 13 | 13 | 13 |
| **Maximum score possible** |  | 19 | 19 | 19 | 14 | 19 | 19 | 14 | 14 | 14 | 19 | 14 | 14 | 14 | 14 | 14 |
| **Percentage score (%)** |  | 89.4 | 84.2 | 84.2 | 85.7 | 94.7 | 73.7 | 85.7 | 92.9 | 92.9 | 89.4 | 78.6 | 85.7 | 92.9 | 92.9 | 92.9 |
